# Supplementary material for: Structural variation in nebulin and its impact on phenotype and inheritance: establishing a dominant distal phenotype caused by large deletions
Source: Eur J Hum Genet. 2025 Jun 14;33(9):1153–62. doi: 10.1038/s41431-025-01891-0 (PMC12402328; doi:10.1038/s41431-025-01891-0)
Supplement: Supplementary file 1 [file 41431_2025_1891_MOESM1_ESM.docx]

Supplementary file 1


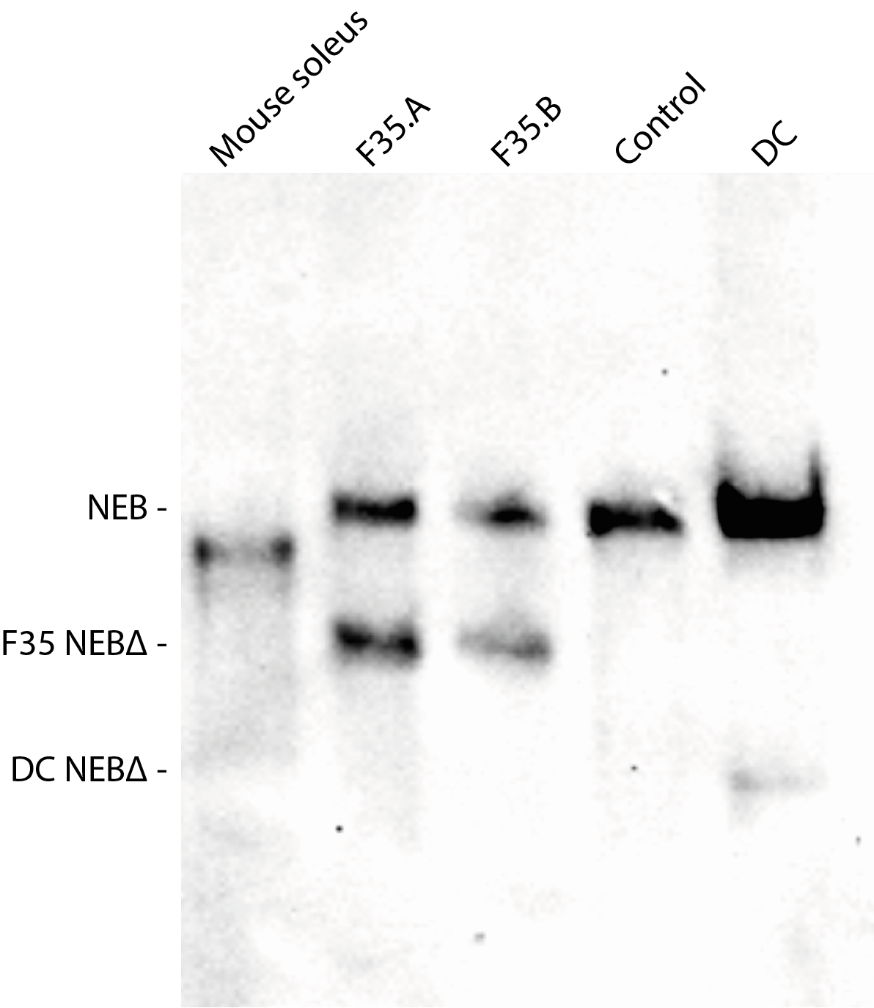


**Supplementary figure 1**. Nebulin western blot analysis performed on proteins extracted from muscle biopsies taken from two affected siblings in F35 shows the corresponding short nebulin isoform (F35 NEBΔ) when detected with an antibody targeting NEB super-repeat 21. The full-size nebulin bands in F35.A, F35.B, Control and the disease control (DC) correspond to a size of 773-986 kDa (Uniprot P20929). The DC NEBΔ isoform is estimated to be 647 kDa. F35.A: individual A in F35 (sibling of F35.B); F35.B: individual B in F35 (sibling of F35.A); NEB: Nebulin; NEBΔ: aberrant nebulin protein; DC: disease control.
